# The Physiological Response of *Salix matsudana* for Water Pollution by 2,4-Dinitrophenol

**DOI:** 10.3390/toxics12100763

**Published:** 2024-10-20

**Authors:** Huicheng Xie, Yikang Fu, Degang Fu, Dengfeng Lin, Huimin Zhou, Guilong Fu, Hui Li, Jinxin Liu, Xiuguo Zheng, Kun Li

**Affiliations:** 1 College of Agriculture and Forestry Science, Linyi University, Linyi 276000, China; xiehc@sdau.edu.cn; 2 College of Forestry, Shandong Agricultural University, Tai’an 271018, China; 15054714947@163.com (H.Z.); fgl009900@163.com (G.F.); kunli@sdau.edu.cn (K.L.); 3 Division of Applied Science, College of International Education, School of Continuing Education, Hong Kong Baptist University, Hong Kong, China; fuyikang_202305@outlook.com; 4 Shandong Provincial Forestry Protection and Development Service Center, Ji’nan 250014, China; 13064030320@163.com; 5 Linyi Forest and Grass Wetland Protection Center, Linyi 276000, China; sdlyldf@126.com; 6 State-Owned Shangting Forest Farm of Shanting County, Zaozhuang 277200, China; liuzi0632@163.com; 7 State-Owned Xuzhuang Forest Farm of Shanting County, Zaozhuang 277217, China; 18266025829@163.com

**Keywords:** 2,4-DNP stress, photosynthesis, phytoremediation, *Salix matsudana*

## Abstract

In this study, the effects of different concentrations of 2,4-dinitrophenol (2,4-DNP) stress on physiological parameters, as well as the uptake and removal of 2,4-DNP in *Salix matsudana*, were investigated using hydroponic simulation experiments to explore the potential of the use of *Salix matsudana* in the phytoremediation of wastewater polluted by 2,4-DNP. The results showed that *P*_N_ (net photosynthetic rate), *T*_r_ (transpiration rate), *G*_s_ (stomatal conductance), *L*_s_ (stomatal limitation value), *F*_v_/*F*_m_ (maximal quantum yield of PSII photochemistry), and *q*_p_ (photochemical quenching coefficient) of *Salix matsudana* seedlings showed an overall decreasing trend, while *C*_i_ (intercellular CO_2_ concentration) showed an increasing trend with the increase in 2,4-DNP concentration. The net photosynthetic rate and intercellular carbon dioxide concentration showed an opposite trend in the leaves with the increase in 2,4-DNP stress concentration, and the inhibition of net photosynthesis by 2,4-DNP on *Salix matsudana* seedlings was mainly based on non-stomatal factors. In the 15 d incubation experiment, the values of SOD (superoxide dismutase), POD (peroxidase), and CAT (catalase) indexes were higher at low concentrations of 2,4-DNP stress, and all three enzymes reached their maximum values at 10 mg L^−1^ of 2,4-DNP and then decreased. *Salix matsudana* seedlings could tolerate 2,4-DNP stress well, which did not exceed 20 mg L^−1^. The toxicity of 2,4-DNP solution was significantly reduced after purification by *Salix matsudana* seedlings. The removal rate of 2,4-DNP was higher than 80% in each treatment group by *Salix matsudana* purified after 15 days. When the concentration of 2,4-DNP reached 20 mg L^−1^, the contents of MDA (malonicdialdehyde) were 55.62 mmol g^−1^, and the values of REC (relative conductivity) and LD (leaf damage) were 63.51% and 59.93%, respectively. The structure and function of the cell membrane in leaves were seriously damaged. With the increase in 2,4-DNP concentration, the removal of 2,4-DNP by *Salix matsudana* seedlings showed a decreasing trend. When the 2,4-DNP concentration was 5 mg L^−1^, the highest removal rate of 2,4-DNP by *Salix matsudana* seedlings was 95.98%, while when the 2,4-DNP concentration was 20 mg L^−1^, the highest removal rate was 86.76%. It is noted that the suitable, recommended concentration for the phytoremediation of 2,4-DNP contamination by *Salix matsudana* seedlings is between 8.81 and 13.78 mg L^−1^.

## 1. Introduction

As an important chemosynthetic fuel among phenolic compounds, nitrobenzene phenolics often follow the discharge of wastewater to pollute the surrounding environment and endanger flora, fauna, microorganisms, and human health during use and production [1]. Among them, 2,4-dinitrophenol (2,4-DNP), as an emerging pollutant, has strong toxicity and slow biodegradation [2,3,4]., it has been included in the “Priority Control Pollutant List” because of its high toxicity [5] Under natural environments, 2,4-DNP has strong stability and a slow rate of degradation, so it persists in the environment for a long time. Therefore, it is essential to remove 2,4-DNP contamination from the environment.

At present, the removal of 2,4-DNP pollutants in the environment is mainly carried out by physical, chemical, and biological methods. However, the physical and chemical methods are costly, cannot achieve complete purification, and are prone to environmental pollution, thus leading to potential ecological problems. The biodegradation rate of conventional activated sludge is usually very low. Phytoremediation technology is in the form of the enrichment, decomposition, and transformation of toxic and harmful pollutants in the environment through the metabolic and uptake functions of plants [6,7]. Jha et al. [8] used sunflower (*Helianthus annuus* L.) trichome roots treated with different concentrations of phenol (100–500 mg L^−1^). The results showed that 100 mg L^−1^ of phenol could be completely removed by the trichome roots of a sunflower after 144 h of treatment. Li et al. [9] studied the effect of phenol on the photosynthetic physiological parameters of *Salix matsudana* leaves, finding that *Salix matsudana* could be applied to the remediation of phenol-contaminated water bodies when the phenol concentration was below 200 mg L^−1^. Dong et al. [10] found that *Potamogeton crispus* and *Nasturium officinale* could remove phenol at low to medium concentrations (≤6 mg L^−1^) in the water column and grow well.

*Salix matsudana*, one of the common willow species, is widely distributed in China and has great potential for the phytoremediation of polluted water bodies or soil environments. Wang et al. [11] studied the effects of Cd uptake and accumulation in *Salix matsudana* under Cd stress. The results showed that *Salix matsudana* could better absorb Cd from the environment, and the Cd content in *Salix matsudana* increased with Cd concentration. Zhu et al. [12] investigated the tolerance, enrichment effect, and transport characteristics of Pb in *Salix matsudana*. The results showed that lower concentrations (≤600 mg kg^−1^) of Pb promoted the growth of *Salix matsudana*, while higher concentrations (≥1000 mg kg^−1^) of Pb had the opposite result. Mohsin et al. [13] used *Salix schwerinii* to purify and remediate phenolic pollutants in soil. Fu et al. [14] found that the ability of *Salix babylonica* to tolerate 2,4-DNP could be effectively enhanced by adding exogenous selenium. Shi et al. [15] found that *Salix matsudana* had a better remediation effect on water polluted by low-concentration 2,4-DCP. Xie et al. [16] found that *Salix matsudana* could remove phenolic compounds from wastewater. However, studies on the effects of 2,4-DNP containing wastewater on the growth and physiology of *Salix matsudana* and the purification effect of *Salix matsudana* on 2,4-DNP containing wastewater have not been reported yet. In this paper, the effect of different concentrations of 2,4-DNP solution on the photosynthesis and growth of *Salix matsudana*, as well as its purification effect, was observed by using hydroponic simulation experiments with *Salix matsudana* seedlings as experimental materials. The aim of this paper was to explore the tolerance of *Salix matsudana* to 2,4-DNP pollution and the feasibility of applying *Salix matsudana* to remediate wastewater polluted by 2,4-DNP.

## 2. Materials and Methods

### 2.1. Experimental Material

Healthy branches from *Salix matsudana* in East-Lake Park (117°15′ E, 36°19′ N), Tai’an, Shangdong, China, were cut in early March. These branches were cut into lengths of 20 cm after being washed with tap water. Each cutting was placed in a 500 mL conical flask for rooting culture at 25 °C. After 15 days, the cuttings continued to be cultured with 1/2 Hoaglands nutrient solution (400 mL), and each conical bottle was wrapped in a black plastic bag to inhibit algal growth.

### 2.2. Experimental Design

Healthy and uniformly growing *Salix matsudana* seedlings were treated with different concentrations of 2,4-DNP stress. Seven treatment groups were set up with 2,4-DNP (Jingchun Reagent Co., Ltd., Shanghai, China) concentrations of 0 (CK), 5, 10, 15, 20, 25, and 30 mg L^−1^, respectively. Photosynthesis, chlorophyll fluorescence parameters, the activity of antioxidant enzymes, the percentage of 2,4-DNP removal, and other indicators of leaves in each treatment group were measured at 5 d, 10 d, and 15 d under 2,4-DNP stress. At the end of the experiment, leaf relative electrical conductivity, malondialdehyde content, relative electrical conductivity, changes in growth morphological characteristics, and the root system were measured.

### 2.3. Experimental Method

#### 2.3.1. Photosynthetic Parameters

Photosynthetic basic indicators were measured with a Li-6800 Photosynthesis System (LI-COR Inc., Lincoln, NE, USA) at photosynthetically active radiation of 1200 μmol m^−2^ s^−1^. The net photosynthetic rate (*P*_N_), transpiration rate (*T*_r_), intercellular CO_2_ concentration (*C*_i_), and stomatal conductance (*G*_s_) were used as basic photosynthetic indicators. The water-use efficiency (WUE), light-energy-use efficiency (LUE), and stomatal limitation value (*L*_s_) were calculated [17].

#### 2.3.2. Chlorophyll Fluorescence Parameters

Well-developed leaves were selected from each seedling undergoing different treatments, and the chlorophyll fluorescence parameters of leaves were measured with a fluorometer (Hansatech, Kings’s Lynn, Norfolk, UK) [18]. Some other Chl fluorescence parameters were calculated according to the equations as follows [19]:

Maximal quantum yield of PSII photochemistry (*F*_v_/*F*_m_) = (*F*_m_ − *F*_o_)/*F*_m_

Photochemical quenching coefficient (*q*_p_) = (*F*_m_*′* − *F*_s_)/(*F*_m_*′* − *F*_o_*′*)

Nonphotochemical quenching (NPQ) = (*F*_m_ − *F*_m_*′*)/*F*_m_*′*

Effective quantum yield of PSII photochemistry (*Φ*_PSII_) = (*F*_m_*′* − *F*_s_)/*F*_m_*′*

*F*_v_*:* variable fluorescence; *F*_m_: maximum fluorescence; *F*_o_: initial fluorescence; *F*_m_*′*: maximum fluorescence under light; *F*_s_: steady-state fluorescence; *F*_o_*′*: minimum fluorescence under light.

#### 2.3.3. Chlorophyll Content

The chlorophyll content of each plant was determined by a spectrophotometer (Epoch2T, Biotek, Vermont, USA). 0.2 g fresh leaves were weighed from each plant [20].

chlorophyll a (Chl*a*) = 13.95A_665_ − 6.88A_649_

chlorophyll b (Chl*b*) = 24.96A_649_ − 7.32A_665_

carotenoids (Car) = (1000A_470_ – 2.05 Chl*a* − 114.8 Chl*b*)/245

Total chlorophyll (Chl*a* + Chl*b*) = Chl*a* content + Chl*b* content

A_665_: the absorbance at 665 nm; A_649_: the absorbance at 649 nm; A_470_: the absorbance at 470 nm

#### 2.3.4. Percentage Removal of 2,4-DNP

The measurement of 2,4-DNP removal was carried out using an enzyme marker (Epoch2T, Biotek, VT, USA). A total of 1 mL of the treatment solution was absorbed, and after high-speed centrifugation at 12,000 r m^−1^ for 3 min. The OD values were measured at 10 nm intervals in the 300–600 nm band and at 360 nm alone. A standard curve was made using the series of 0.0 mg L^−1^, 1.0 mg L^−1^, 2.0 mg L^−1^, 4.0 mg L^−1^, 8.0 mg L^−1^, and 10 mg L^−1^ H_2_O_2_ solution. The standard curve was used to estimate the concentration of 2,4-DNP. The results were measured once every 5 days and repeated 3 times for each treatment. The percentage of 2,4-DNP removal was calculated as follows [21]:

$$P_{T}\;(\%)=\frac{\left(C_{o}-C_{f}\right)}{C_{o}}\times 100\%$$

*P*_T_: percentage removal of 2,4-DNP; *C*_i_: initial concentration of 2,4-DNP (mg L^−1^); *C*_f_: final concentration of 2,4-DNP (mg L^−1^).

#### 2.3.5. Antioxidant Enzyme Activity and MDA Content

The phosphoric acid buffer (1 mL, 0.05 mol L^−1^, pH 7.8, Macklin Biochemical Technology Co., Ltd., Shanghai, China) was injected into fresh weight leaves (0.2 g) for ice bath grinding. Then, the phosphate buffer was added to the obtained grinding solution (4 mL, Macklin Biochemical Technology Co., Ltd., Shanghai, China) after pouring it into the centrifuge tube. After high-speed centrifugation [22], the supernatant was extracted for antioxidant enzyme activity and MDA content determination.

The activities of peroxidase (POD) and superoxide dismutase (SOD) were determined by the method of Ying et al. [23]. The activities of catalase (CAT) and malondialdehyde content (MDA) were determined using the method in the literature [24].

#### 2.3.6. Relative Electrical Conductivity (REC)

The relative conductivity was determined using the method according to Zhong et al. [25].

REC (%) = (S_1_ − S_0_) × 100/(S_2_ − S_0_)

S_0_: blank conductivity; S_1_: initial conductivity; S_2_: final conductivity.

### 2.4. Data Analysis

The analytical calculations of experimental data were carried out using SPSS 22.0 software (IBM, Chicago, IL, USA). The mean value was analyzed by one-way ANOVA, and the difference in mean was compared by Duncan at the 0.05 confidence level. Plotting was used by Origin 9.0 (OriginLab, Northampton, Massachusetts, USA).

## 3. Results

### 3.1. Photosynthetic Gas Exchange Parameters

**Figure 1:**
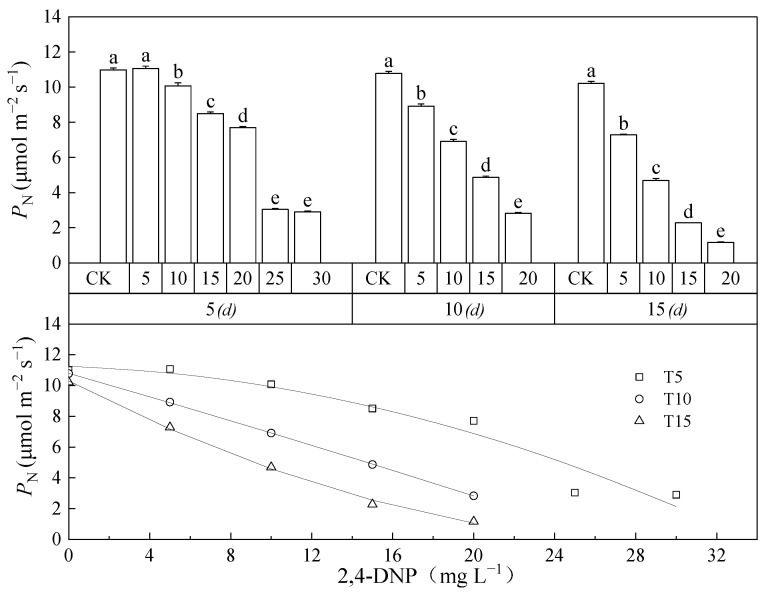
The *P*_N_ of *Salix matsudana* seedlings leaves changes with concentration and time under stress of 2,4-DNP and its unary quadratic equation simulation. Note: different lowercase letters indicate that the *P*_N_ of the leaves of *Salix matsudana* seedlings was significantly different at the same time between different concentrations of 2,4-DNP (*p* < 0.05).

The net photosynthetic rate (*P*_N_) of the leaves of *Salix matsudana* seedlings treated with different concentrations of 2,4-DNP stress at 5, 10, and 15 d changed significantly (*p* < 0.05), and the decrease in *P*_N_ became more pronounced with time (Figure 1).

As shown in Figure 1, with the increase in treatment time and concentration of 2,4-DNP stress, the *P*_N_ of *Salix matsudana* leaves was inhibited more seriously, while its photosynthetic capacity decreased gradually. At the same concentration (20 mg L^−1^) of 2,4-DNP stress, the *P*_N_ of *Salix matsudana* leaves was 7.70 μmol m^−2^ s^−1^ after 5 d, 2.83 μmol m^−2^ s^−1^ after 10 d, and 1.17 μmol m^−2^ s^−1^ after 15 d in *Salix matsudana* leaves, which were 70.1%, 25.8%, and 10.66% of the control values, respectively. The equations fitted to them by quadratic equations were Y = −0.0085X^2^ – 0.0491X + 11.257, Y = −0.0011X^2^ − 0.3765X + 10.792, and Y = 0.0109X^2^ − 0.6802X + 10.295, respectively. The concentrations of 2,4-DNP at when *P*_N_ was halved were calculated to be 23.01, 13.78 and 8.81 mg L^−1^, so it could be determined that the tolerance thresholds of *Salix matsudana* seedlings to 2,4-DNP at 5, 10 and 15 d of 2,4-DNP stress were 23.01, 13.78 and 8.81 mg L^−1^, respectively.

**Figure 2:**
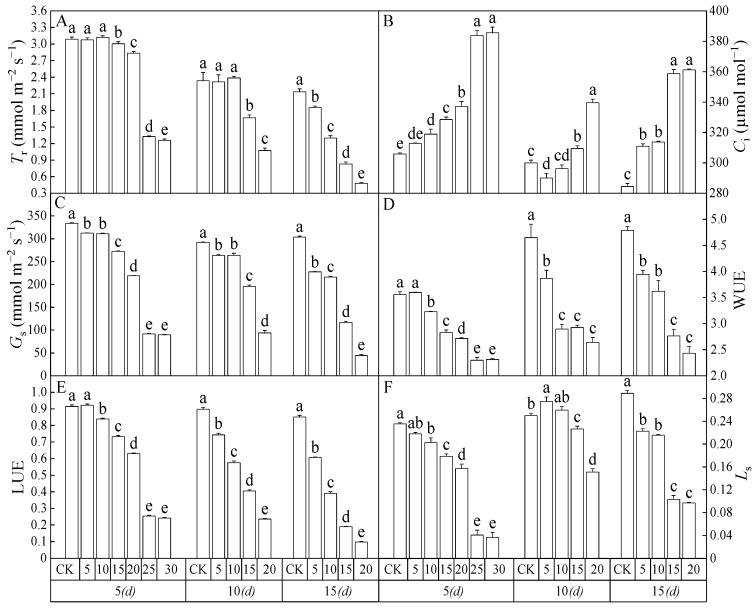
The photosynthetic gas exchange parameters of the leaves of *Salix matsudana* seedlings under the stress of 2,4-DNP changed with concentration and time. (**A**) Transpiration rate (*T*_r_); (**B**) Intercellular CO_2_ concentration (*C*_i_); (**C**) Stomatal conductance (*G*_s_); (**D**) water-use efficiency (WUE); (**E**) light-energy-use efficiency (LUE); (**F**) Stomatal limitation value (*L*_s_). Note: Different lowercase letters within different treatments are significantly different at the same time (*p* < 0.05).

As shown in Figure 2, the *T*_r_, *P*_N_, *G*_s_, *L*_s_, *C*_i_, WUE, and LUE of *Salix matsudana* seedlings treated with different concentrations of 2,4-DNP for 5, 10 and 15 d were significantly different from each other (*p* < 0.05). *C*_i_, WUE, and LUE varied significantly (*p* < 0.05) from each other. After 5 d, with the increase in the 2,4-DNP concentration, the *P*_N_, *T*_r_, LUE, and WUE of *Salix matsudana* seedling leaves showed increasing and then decreasing trends (Figure 1 and Figure 2A,D,E), both *G*_s_ and Ls showed decreasing trends (Figure 2C,F), and *C*_i_ showed an increasing trend (Figure 2B). At 10and 15 d, with the increase in 2,4-DNP concentration, the leaf *C*_i_ of *Salix matsudana* seedlings showed an increasing trend (Figure 2B), except for the other indicators, which showed a decreasing trend (Figure 1 and Figure 2). After 10 d, when the concentration of 2,4-DNP increased to 25 mg L^−1^ and 30 mg L^−1^, the seedlings of *Salix matsudana* showed a loss of physiological activity and died; therefore, their related indexes were not measured. This result indicated that 25 mg L^−1^ of 2,4-DNP exceeded the tolerance limit of *Salix matsudana* seedlings themselves under 2,4-DNP stress treatment within 10 d. After 15 days, *T*_r_, *G*_s_, WUE, LUE, and *L*_s_ were reduced to the lowest values, which were 22.49%, 14.58%, 33.53%, 11.41%, and 50.81% of the control (CK) values, respectively.

### 3.2. Chlorophyll Fluorescence Parameters

**Figure 3:**
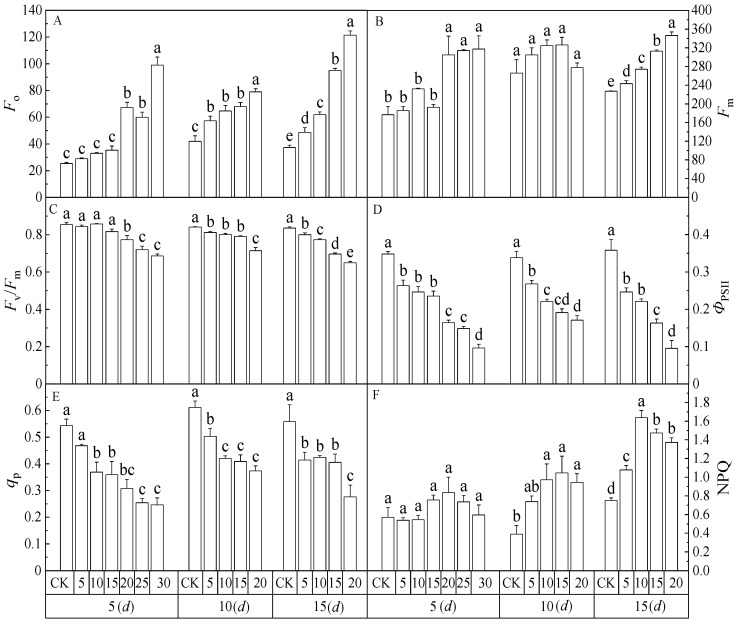
Changes in the chlorophyll fluorescence parameters of leaves of *Salix matsudana* seedlings with 2,4-DNP concentrations and stress times. (**A**) Including initial fluorescence (*F*_O_); (**B**) Maximum fluorescence (*F*_m_); (**C**) Maximal quantum yield of PSII photochemistry (*F*_V_/*F*_m_); (**D**) Effective quantum yield of PSII photochemistry (*Φ*_PSII_); (**E**) Photochemical quenching coefficient (*q*_p_); (**F**) Nonphotochemical quenching (NPQ). Note: Different lowercase letters indicate that the chlorophyll fluorescence parameters of the leaves of *Salix matsudana* seedlings were significantly different at the same time between different concentrations of 2,4-DNP (*p* < 0.05).

As shown in Figure 3, the *F*_o_, *F*_m_, *F*_v_/*F*_m_, *q*_p_, NPQ, and *Φ*_PSII_ of *Salix matsudana* seedling leaves showed significant differences overall with increasing 2,4-DNP concentration (*p* < 0.05). Among them, *F*_o_ and *F*_m_ showed increasing trends, *F*_v_/*F*_m_, *q*_p_, and *Φ*_PSII_ showed decreasing trends, and NPQ showed an increasing and then decreasing trend. For NPQ, with the increase in 2,4-DNP concentration, which has no significant difference on the 5th day. The NPQ of leaves showed an increasing trend on the 10th day, and an increasing and then decreasing trend on the 15th day. Therefore, the distribution and utilization of light energy of *Salix matsudana* were regulated by non-photochemical energy dissipation.

### 3.3. Chlorophyll Content

**Figure 4:**
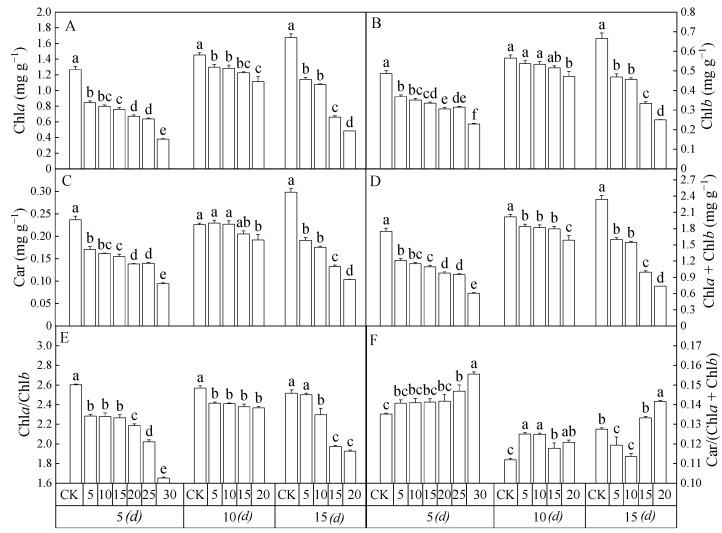
The chlorophyll contents of the leaves of *Salix matsudana* seedlings under the stress of 2,4-DNP. (**A**) chlorophyll a (Chl*a*); (**B**) chlorophyll b (Chl*b*); (**C**) carotenoid (Car); (**D**) chlorophyll a + chlorophyll b (Chl*a* + Chl*b*); (**E**) chlorophyll a/chlorophyll b (Chl*a*/Chl*b*); (**F**) carotenoid/(chlorophyll a + chlorophyll b) (Car/(Chl*a* + Chl*b*)). Note: Different lowercase letters indicate that the chlorophyll content of the leaves of *Salix matsudana* seedlings was significantly different at the same time between different concentrations of 2,4-DNP treatments (*p* < 0.05).

As shown in Figure 4, the chlorophyll content of the leaves of *Salix matsudana* seedlings showed trends of increasing and then decreasing with time. During days 0–10 of stress, the Chl*a*, Chl*b*, Car, and total chlorophyll content (Chl*a* + Chl*b*) of seedling leaves in all treatment groups gradually increased, chlorophyll a/chlorophyll b (Chl*a*/Chl*b*) remained unchanged, and carotenoids/total chlorophyll content (Car/(Chl*a* + Chl*b*)) slightly decreased. Within 10–15 d, Chl*a*, Chl*b*, Car, Chl*a* + Chl*b* and Chl*a*/Chl*b* of seedling leaves in all treatment groups showed a decreasing trend except for the control group (CK), and Car/(Chl*a* + Chl*b*) remained unchanged. With the increase in 2,4-DNP concentration, the Chl*a*, Chl*b*, Car, Chl*a* + Chl*b* and Chl*a*/Chl*b* of the seedling leaves in all treatment groups gradually decreased, except Car/(Chl*a* + Chl*b*). The results showed that 2,4-DNP stress inhibited chlorophyll synthesis or accelerated the chlorophyll degradation of *Salix matsudana* seedling leaves, and the higher the concentration of 2,4-DNP, the stronger the damage to chlorophyll in the leaves of *Salix matsudana* seedlings.

### 3.4. Level of Cell Membrane Damage

As shown in Table 1, the MDA, REC, and LD of *Salix matsudana* seedling leaves showed a significant upward trend with increasing 2,4-DNP concentration (*p* < 0.05). At 2,4-DNP concentrations of 5, 10, 15, and 20 mg L^−1^, respectively, the MDA of *Salix matsudana* seedling leaves was 141.98%, 206.99%, 343.67%, and 475.48% of the control (CK) values. REC was 174.16%, 264.68%, 559.50%, and 710.33% of the control group (CK) values, respectively. LD was 7.28%, 16.18%, 45.11%, and 59.93%, respectively, with the latter increasing by 122.14%, 178.89%, and 32.85%, over the former. It can be seen that when the concentration of 2,4-DNP was in the range of 10–15 mg L^−1^, the structure of the cell membrane was damaged, but the function of the cell membrane was still relatively stable in the leaves of *Salix matsudana* seedlings. When the concentration of 2,4-DNP was 20 mg L^−1^, MDA, REC, and LD increased to 55.62 mmol g^−1^, 63.51%, and 59.93%, respectively, and the structure and function of the cell membrane in the leaves were seriously damaged.

### 3.5. Antioxidant Enzyme Activity

As shown in Table 2, the activities of CAT, POD, and SOD in the leaves of *Salix matsudana* seedlings showed significant differences with increasing concentrations of 2,4-DNP (*p* < 0.05). At 5 d, POD and CAT reached their extreme values at a 2,4-DNP concentration of 20 mg L^−1^ and then decreased to 700.00% and 460.00% of the CK value, respectively, while SOD reached its extreme value at a 2,4-DNP concentration of 15 mg L^−1^ and then decreased to 759.68% of the CK value. At 10 d, except for POD, which showed an increasing trend, SOD and CAT both reached extreme values at a 2,4-DNP concentration of 15 mg L^−1^ and then declined to 656.99% and 316.67% of the CK value, respectively. At 15 d, all three enzymes reached maximum values at a 2,4-DNP concentration of 10 mg L^−1^ and then decreased to 438.72%, 371.88%, and 237.50% of the CK values, respectively. It could be seen that under 2,4-DNP stress, the antioxidant system of the leaves of *Salix matsudana* seedlings was activated to generate the corresponding protective mechanism to cope with the external stress environment by increasing the activities of CAT, POD, and SOD, thus playing a protective role for themselves.

### 3.6. Reactive Oxygen Species Level

**Figure 5:**
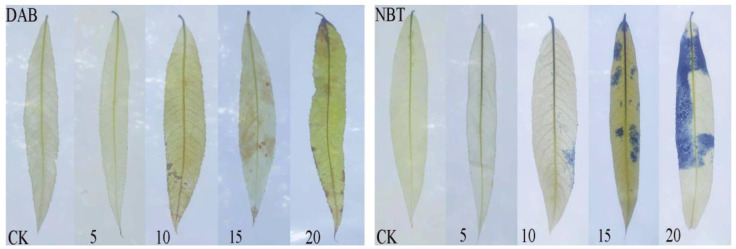
In situ histochemical detection of hydrogen peroxide (H_2_O_2_) and superoxide (O^2−^∙) using DAB and NTB staining, respectively.

The histochemical staining of H_2_O_2_ and O_2_^−^∙ in leaves of *Salix matsudana* seedlings revealed the accumulation of H_2_O_2_ and O_2_^−^∙ in the leaves (Figure 5). On the 15th day, the accumulation of H_2_O_2_ and O_2_^−^∙ in each treatment group under 2,4-DNP stress was obviously higher than that in its CK group. Black-brown (DBA) and visible dark-blue (NBT) spots appeared on the leaves, and the amount increased with the increase in 2,4-DNP concentration.

**Figure 6:**
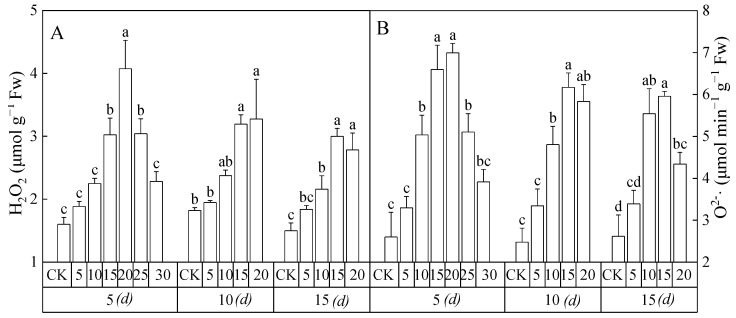
The ROS levels of the leaves of *Salix matsudana* seedlings under the stress of 2,4-DNP changed with concentration and time. (**A**) Hydrogen peroxide; (**B**) Superoxide radical. Note: Different lowercase letters indicate that the ROS levels of the leaves of *Salix matsudana* seedlings were significantly different at the same time between different concentrations of 2,4-DNP treatments, at *p* < 0.05.

The contents of H_2_O_2_ and O_2_^−^∙ in the leaves of *Salix matsudana* seedlings showed significant differences with the increase in 2,4-DNP concentration and stress time (Figure 6). On the 5th day, the content of H_2_O_2_ and O_2_^−^∙showed trends of increasing first and then decreasing with the increase in 2,4-DNP concentration. When the concentration of 2,4-DNP was 20 mg L^−1^, the contents of H_2_O_2_ and O_2_^−^∙ reached their maximum values, which were 1.69 times and 1.55 times higher than those of the CK group, respectively. On the 10th day, the content of H_2_O_2_ increased with the increase in 2,4-DNP concentration. Among them, when the concentration of 2,4-DNP was 5 mg L^−1^, 10 mg L^−1^, 15 mg L^−1^ and 20 mg L^−1^, the content of H_2_O_2_ was 1.07 times, 1.30 times, 1.75 times and 1.80 times that of the CK group value, respectively. On the 15th day, the content of H_2_O_2_ and O_2_^−^∙ reached the maximum value at the concentration of 15 mg L^−1^ of 2,4-DNP, which were 2.00 times and 2.27 times that of the CK group, respectively. However, there was no significant difference in the contents of H_2_O_2_ and O_2_^−^∙ at 2,4-DNP concentrations of 15 mg L^−1^and 20 mg L^−1^.

### 3.7. 2,4-DNP Removal Effect

**Figure 7:**
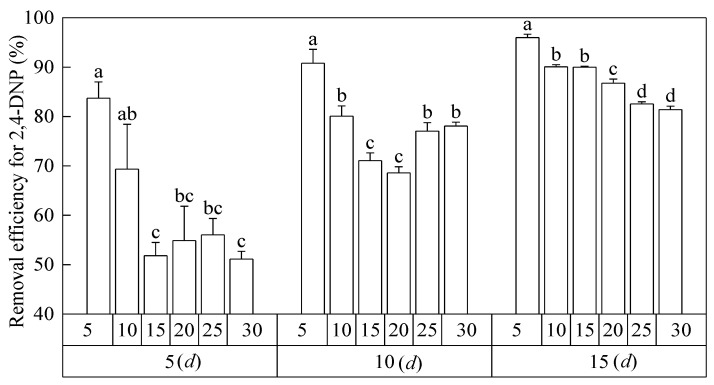
The percentage of 2,4-DNP removal of *Salix matsudana* seedlings under the stress of 2,4-DNP changed with concentration and time. Note: Different lowercase letters indicate that the percentage of 2,4-DNP removal of *Salix matsudana* seedlings was significantly different at the same time between different concentrations of 2,4-DNP, at *p* < 0.05.

As shown in Figure 7, the percentage removal of 2,4-DNP in the water column showed a gradual increase with the increase in treatment time of 2,4-DNP in the water column. After 5 d of the purification of *Salix matsudana* seedlings, the percentage removal of 2,4-DNP from water column was 83.70%, 69.33%, 51.81%, 54.86%, 56.02%, and 51.12% with the increase in 2,4-DNP concentration. After 10 d of purification of *Salix matsudana* seedlings, the percentage removal of 2,4-DNP was 90.80%, 80.07%, 71.05%, 68.58%, 77.05%, and 78.07% with increasing 2,4-DNP concentrations, respectively. After 15 d of the purification of *Salix matsudana* seedlings, the percentage removal of 2,4-DNP was 95.98%, 90.06%, 89.99%, 86.76%, 82.55%, and 81.39% with increasing 2,4-DNP concentration. At 10 and 15 d, the seedlings of *Salix matsudana* at 2,4-DNP concentrations of 25 and 30 mg L^−1^ died.

## 4. Discussion

It was reported that *Salix matsudana* seedlings are mostly used for phytoremediation, as well as on river banks and degraded polluted soil [26,27]. *Salix matsudana* seedlings could reduce the pollution of wastewater from dairy farms and do not affect the local water system [28]. The normal growth of plants could be affected by adversity stress, and photosynthesis is the basis for the normal growth and development of plants [29]. The factors that usually affect plant photosynthesis are divided into stomatal and non-stomatal factors [30]. The former refers to the decrease in stomatal conductance due to water stress and the decrease in photosynthesis due to blocked CO_2_ entry into the leaf, while the latter refers to the decrease in photosynthetic activity of the chloroplast [31,32]. Li et al. [8] found that the net photosynthesis of *Salix matsudana* showed a decreasing trend at a low phenol concentration (50 mg L^−1^). Vilyanen et al. [33] found that a 2,4-dinitrophenyl ether of 2-iodo-4-nitrothymol could significantly reduce the photosynthesis of plants by inhibiting photosynthetic electron transport. Jing et al. [34] found that an aniline concentration greater than 200 mg L^−1^ inhibited the growth of sunflowers. The above studies have shown that the toxicity of organic matter to plants varies widely and that the photosynthetic physiological response of plants to organic pollution stress also exhibits variability. In this study, *P*_N_ and *G*_s_ of *Salix matsudana* seedling leaves showed an overall decreasing trend with increasing 2,4-DNP concentration, while *C*_i_ showed an overall increasing trend. In this study, the seedlings of *Salix arundinacea* had some defensive behaviors to deal with the low concentration of 2,4-DNP, but when the concentration and time of 2,4-DNP stress were further increased, beyond the tolerance limit of *Salix arundinacea*, the photosynthetic capacity of its leaves was severely inhibited. With the increase in 2,4-DNP concentration, the *P*_N_ of *Salix matsudana* seedlings decreased, while *C*_i_ increased. According to Farquhar and Sharkey’s criterion of photosynthesis restriction [35], the main reason for the photosynthesis decrease in *Salix matsudana* seedlings caused by 4-DNP was the limitation of non-stomatal factors. There should be a critical concentration of 2,4-DNP between 8.81 and 13.78 mg L^−1^ for *Salix matsudana* seedlings to transition from stomatal limiting factors to non-stomatal limiting factors in the 15 d treatment. The specific value needs further study.

Chlorophyll fluorescence parameters respond to the absorption, distribution, dissipation, and transfer of light energy by the photosystem during photosynthesis in plants [36]. *F*_o_ is the fluorescence level when the PSII reaction center is fully open, and *F*_m_ reflects the degree of electron transfer and photoinhibition of the PSII reaction center, and its value size indicates the higher degree of disruption or reversible inactivation of the PSII reaction center [37]. In this study, *F*_o_ and *F*_m_ increased with increasing 2,4-DNP concentration. The difference between a 2,4-DNP concentration of higher than 20 mg L^−1^ and the remaining control reached a significant level, indicating that a high concentration of 2,4-DNP could cause the destruction or reversible inactivation of PSII reaction center. *F*_v_/*F*_m_ is the maximum photochemical quantum yield of PSII, which reflects the light energy conversion efficiency of PSII reaction centers. Generally, the *F*_v_/*F*_m_ size of higher plants under normal conditions is between 0.80 and 0.84 [38]. In this study, after 2,4-DNP was greater than 10 mg L^−1^ in the 15 d purification experiment, the *F*_v_/*F*_m_ value gradually decreased with increasing concentration to 0.65 at 20 mg L^−1^, indicating that photosynthesis in the leaves of *Salix matsudana* seedlings was subject to photoinhibition, or the PSII system was damaged at this time. *Φ*_PSII_ reflects the ratio of light energy absorbed by photosynthetic bodies for photochemical reactions under light, *q*_p_ reflects the number of PSII open centers in plant leaves, and larger values indicate higher electron transfer activity of PSII in plant leaves [39]. In this experiment, with the increase in 2,4-DNP concentration, both *q*_p_ and *Φ*_PSII_ showed decreasing trends, indicating that 2,4-DNP stress would increase the degree of closure of the PSII reaction center, decrease the photosynthetic electron transfer rate, and decrease the energy used for the actual chemical reaction of the photosystem. NPQ represents the portion of light energy absorbed by the antenna pigments of PSII that are not used for photosynthetic electron transfer but are dissipated in the form of thermal dissipation, which reflects the ability of the plant leaf photosystem to dissipate excess light energy and facilitates the plant’s protection of its organs [40]. In this study, with the increase in 2,4-DNP concentration, NPQ increased from 0.75 in the control group (CK) to 1.64 at 10 mg L^−1^ and then gradually decreased to 1.37 at 20 mg L^−1^, with an overall trend of increasing and then decreasing. Meanwhile, *F*_0_ continued to increase all the time, indicating that the energy used for the thermal dissipation of antenna pigments in *Salix matsudana* seedlings under 10 mg L^−1^ 2,4-DNP stress was reduced. The reason for this might be that high concentrations of 2,4-DNP (>15 mg L^−1^) had a certain degree of effect on the violet xanthin dehydrogenase in their bodies, resulting in reduced thermal dissipation dependent on the lutein cycle. The PSII photosynthetic system of *Salix matsudana* seedlings was significantly damaged by strong light [41], which was similar to the mechanism through which high concentrations of phenolic acids degraded photosynthesis in poplar [42].

When plants are subjected to external stress, their reactive oxygen species (ROS) are produced substantially to enhance metabolism and self-resistance [43,44]. When large amounts of ROS cannot be removed promptly, it will cause metabolic disorders in plants and weaken normal physiological activities, leading to plant death in severe cases [45,46]. At 2,4-DNP concentrations of 0–10 mg L^−1^, the activities of CAT, POD, SOD, MDA, REC, and LD increased significantly in the leaves of *Salix matsudana* seedlings. The leaves of *Salix matsudana* seedlings at 25–30 mg L^−1^ lost their green color and died over days 5–10. At 10–15 d, REC and LD increased significantly at 2,4-DNP concentrations of 20 mg L^−1^. The high concentration of 2,4-DNP stress increased the damage to the cell membrane structure of the leaves of *Salix matsudana* seedlings, resulting in a large amount of electrolyte exudation and serious cell membrane function. It could be seen that the antioxidant mechanism of the leaves of *Salix matsudana* seedlings under mild 2,4-DNP stress was turned on. The activity of protective enzymes in their bodies was enhanced, which effectively scavenged reactive oxygen species through the synergistic effect of SOD and POD to reduce the damage caused by 2,4-DNP stress to their membrane system and prevent damage to *Salix matsudana* seedlings. When the concentration of 2,4-DNP increased, the activity of SOD and POD gradually decreased and exceeded their thresholds for scavenging reactive oxygen species, and the content of O_2_^−^∙ and H_2_O_2_ generated by SOD increased. This led to an increase in membrane lipid peroxidation damage products (MDA) and an increase in REC and LD, thus causing damage to the leaves of *Salix matsudana* seedlings.

The mortality rate of plants is used as an important indicator for the detection of harmful substances. In this study, the death of *Salix matsudana* seedlings occurred when the 2,4-DNP concentration was 25 mg L^−1^, indicating that 25 mg L^−1^ 2,4-DNP had reached the maximum tolerance limit of *Salix matsudana*. Zhang et al. [47] found that the percentage removal of aniline (100 mg L^−1^) by *Salix matsudana* seedlings could reach 88.6%. In this study, the removal of 2,4-DNP by *Salix matsudana* was 95.98%, 90.06%, 89.99%, 86.76%, 82.55%, and 81.39% at 2,4-DNP concentrations of 5, 10, 15, 20, 25, 30 mg L^−1^ on day 15, respectively. Low concentrations of 2,4-DNP (<10 mg L^−1^) stress could cause microbial cells to secrete large amounts of extracellular polymeric substances, while when the concentration of 2,4-DNP further increases (>10 mg L^−1^), their extracellular polymer production will sharply decrease [47]. In this study, dry willow seedlings showed a good removal effect (>90.06%) of 2,4-DNP at low concentrations (<10 mg L^−1^), but the percentage of 2,4-DNP removal gradually decreased with the increase in 2,4-DNP concentration. Therefore, it was speculated that there was some microbial action involved in the removal of 2,4-DNP by dry willow seedlings, which produced some of the extracellular polymers and extracellular enzymes such as peroxidase.

In this study, the photosynthesis, antioxidant enzyme activity, cell membrane damage level, active oxygen levels, and the 2,4-DNP removal effect were analyzed to elucidate the tolerance range, purification effect, and regulatory effect of *Salix matsudana* on 2,4-DNP in wastewater. However, the important values and correlations among these indicators, as well as the stress resistance index and root index of *Salix matsudana*, need to be further studied.

## 5. Conclusions

The photosynthesis of *Salix matsudana* decreased significantly with the increase in 2,4-DNP concentration. The inhibition of net photosynthesis by 2,4-DNP on *Salix matsudana* seedlings was mainly based on non-stomatal factors. A total of 25 mg L^−1^ of 2,4-DNP exceeded the tolerance limit of *Salix matsudana* seedlings themselves under 2,4-DNP stress treatment within 10 d. After 15 days, the *T*_r_, *G*_s_, WUE, LUE, and *L*_s_ of *Salix matsudana* seedlings were reduced to the lowest values. Under 2,4-DNP stress, the antioxidant system was activated to generate the corresponding protective mechanism to cope with the external stress environment by increasing the activities of CAT, POD, and SOD, thus playing a self-protective role. At 10 and 15 d, the seedlings of *Salix matsudana* at 2,4-DNP concentrations of 25 and 30 mg L^−1^ died. The high concentration of 2,4-DNP stress severely damaged the antioxidant system activity and inhibited the growth of *Salix matsudana* seedlings, leading to a decrease in the percentage of 2,4-DNP removal. Simulation studies showed that the best concentration for the phytoremediation of 2,4-DNP contamination in *Salix matsudana* seedlings was between 8.81 and 13.78 mg L^−1^ to avoid significantly inhibiting the normal growth and remediation effect of *Salix matsudana* seedlings. The research results could provide a theoretical reference for the wastewater remediation of *Salix matsudana*.

## Figures and Tables

**Table 1:** 2,4-DNP content on the malondialdehyde (MDA), relative conductivity (REC) of leaf, and leaf damage (LD) of the leaves of *Salix matsudana* seedlings.

2,4-DNP Concentration	MDA	REC	LD
CK	11.70 ± 0.60 d	8.94 ± 0.45 e	-
5	16.61 ± 1.07 d	15.57 ± 0.48 d	7.28 ± 0.20 d
10	24.21 ± 2.13 c	23.67 ± 1.88 c	16.18 ± 1.88 c
15	40.20 ± 2.74 b	50.03 ± 0.87 b	45.11 ± 1.06 b
20	55.62 ± 2.93 a	63.51 ± 2.27 a	59.93 ± 2.46 a
25	death	death	death
30	death	death	death

Note: Different lowercase letters indicate that the effect of different 2,4-DNP concentrations on the MDA, REC of leaf, and LD of *Salix matsudana* seedlings was significantly different (*p* < 0.05).

**Table 2:** The antioxidant enzyme activity of the leaves of *Salix matsudana* seedlings under the stress of 2,4-DNP changed with concentration and time.

Time(d)	2,4-DNP Concentration	POD	SOD	CAT
5	CK	833 ± 88.2 d	187.9 ± 14.3 c	16.7 ± 1.53 e
	5	2100 ± 251.7 c	616.8 ± 26.2 b	30.0 ± 2.77 de
	10	3367 ± 233.3 b	813.6 ± 79.2 b	53.3 ± 5.16 bc
	15	5334 ± 185.6 a	1427.5 ± 117.2 a	66.7 ± 6.18 ab
	20	5835 ± 189.1 a	1393.7 ± 115.8 a	76.7 ± 6.32 a
	25	3829 ± 218.6 b	839.9 ± 29.8 b	50.0 ± 4.71 c
	30	3331 ± 185.6 b	621.5 ± 49.8 b	43.3 ± 4.21 cd
10	CK	1200 ± 173.2 d	159.6 ± 15.0 d	20.0 ± 1.96 c
	5	2467 ± 193.0 c	565.9 ± 60.6 c	36.7 ± 3.46 bc
	10	3600 ± 264.6 b	744.2 ± 35.2 b	60.0 ± 5.77 a
	15	4633 ± 202.8 a	1048.3 ± 53.7 a	63.3 ± 5.83 a
	20	4800 ± 264.6 a	853.7 ± 55.9 b	53.3 ± 4.82 ab
15	CK	1067 ± 88.2 c	202.6 ± 13.2 c	26.7 ± 2.19 c
	5	2700 ± 250.9 b	664.0 ± 32.3 b	46.7 ± 3.16 b
	10	3967 ± 202.8 a	888.8 ± 34.4 a	63.3 ± 5.14 a
	15	3733 ± 314.4 ab	848.7 ± 48.0 a	53.3 ± 4.18 ab
	20	3667 ± 338.3 ab	696.9 ± 19.7 b	43.3 ± 3.09 b

Note: Different lowercase letters indicate that the same enzyme activity of the leaves of *Salix matsudana* seedlings was significantly different at the same time between different concentrations of 2,4-DNP, at *p* < 0.05.

## Data Availability

Data are contained within the article.

## Nomenclature

Symbols	Name	Unit
*P*_N_	Net photosynthetic rate	μmol m^−2^ s^−1^
*T*_r_	Transpiration rate	mmol m^−2^ s^−1^
*C*_a_	Atmosphere CO_2_ concentration	μmol mol^−1^
*C*_i_	Intercellular CO_2_ concentration	μmol mol^−1^
*G*_s_	Stomatal conductance	mmol m^−2^ s^−1^
*L*_s_	Stomatal limitation value	%
WUE	Water-use efficiency	-
LUE	Light-energy-use efficiency	-
SOD	Superoxide dismutase	U g^−1^ FW
POD	Peroxidase	U g^−1^ min^−1^
CAT	Catalase	U g^−1^ min^−1^
MDA	Malonicdialdehyde	mmol g^−1^
REC	Relative conductivity	%
LD	Leaf damage	%
*F*_o_	Initial fluorescence	-
*F*_o_ *′*	Minimum fluorescence under light	-
*F*_m_	Maximal fluorescence	-
*F*_m_ *′*	Variable fluorescence under light	-
*F*_v_	Variable fluorescence	-
*F*_s_	Steady-state fluorescence	-
*F*_v_ */F*_m_	Maximal quantum yield of PSII photochemistry	-
*Φ*_PSII_	Effective quantum yield of PSII photochemistry	-
*q*_p_	Photochemical quenching coefficient	-
NPQ	Nonphotochemical quenching	-
Chl*a*	Chlorophyll a	mg g^−1^
Chl*b*	Chlorophyll b	mg g^−1^
Car	Carotenoids	mg g^−1^
Chl*a* + Chl*b*	Total chlorophyll content	mg g^−1^
Chl*a*/Chl*b*	Chlorophyll a/chlorophyll b	-
Car/(Chl*a* + Chl*b*)	Carotenoids/total chlorophyll content	-
*P*_T_	Percentage removal of 2,4-DNP	%
*C_o_*	Initial concentration of 2,4-DNP	mg L^−1^
*C*_f_	Final concentration of 2,4-DNP	mg L^−1^
O_2_^−^∙	Superoxide radical	μmol min^−1^ g^−1^ FW
H_2_O_2_	Hydrogen peroxide	μmol g^−1^ FW

## References

[1] Zhang Y.X., Liu Z.L., Tian P.W. (2013). Research progress on plant effects of nitroaromatic compounds. J. Ecol. Environ..

[2] Dehghanifard E., Jafari A.J., Kalantary R.R., Mahvi A.H., Faramarzim A., Esrafili A. (2013). Biodegradation of 2,4-dinitrophenol with laccase immobilized on nano-porous silica beads. Iran. J. Environ. Healt..

[3] Tang T., Yue Z., Wang J., Chen T.H., Qing C.S. (2017). Goethite promoted biodegradation of 2,4-dinitrophenol under nitrate reduction condition. J. Hazard. Mater..

[4] Rahmani H., Lakzian A., Karimi A., Halajnia A. (2020). Efficient removal of 2,4-dinitrophenol from synthetic wastewater and contaminated soil samples using free and immobilized laccases. J. Environ. Manag..

[5] Jian Y., Wang L., Fu P.P., Yu H. (2004). Photomutagenicity of 16 polycyclic aromatic hydrocarbons from the US EPA priority pollutant list. Mutat. Res.-Gen. Tox. En..

[6] Chandanshive V.V., Kadam S.K., Khandare R.V., Kurade M.B., Jeon B.H., Jadhav J.P., Govindwar S.P. (2018). In situ phytoremediation of dyes from textile wastewaterusing garden ornamental plants, effect on soil quality and plant growth. Chemosphere.

[7] Gerhardt K.E., Gerwing P.D., Greenberg B.M. (2017). Opinion: Taking phytoremediation from proven technology to accepted practice. Plant Sci..

[8] Jha P., Jobby R., Kudale S., Modi N., Dhaneshwar A., Desai N. (2013). Biodegradation of phenol using hairy roots of *Helianthus annuus* L. Int. Biodeter. Biodegr..

[9] Li H., Zhang G.C., Xie H.C., Xu J.W., Li C.R., Sun J.W. (2015). The effects of the phenol concentrations on photosynthetic parameters of *Salix babylonica* L. Photosynthetica.

[10] Dong X.L., Feng J., Xie S.L. (2013). Removal effect of low concentration phenol in water by *Potamogeton crispus* and *Nasturium officinale*. Sci. Technol. Innov. Her..

[11] Wang X.H., Wang S.J., Chen Z., Gong B., Wang X.F., Win M., Shi Q.H., Li Y., Yang F.J. (2016). Effects of exogenous polyamines on nitrate tolerance in cucumber. Russ. J. Plant Physl..

[12] Zhu J., Wang P., Ji S.S., Li M., Lui M.J. (2016). Pb tolerance, enrichment, translocation and stress response of *Salix matsudana* seedlings (*Salix matsudana* kids). J. Environ. Sci..

[13] Mohsin M., Kuittinen S., Salam M.M.A., Peraniemi S., Laine S., Pulkkinen P., Kaipiainen E., Vepsalainen J., Pappinen A. (2019). Chelate-assisted phytoextraction: Growth and ecophysiological responses by *Salix schwerinii* E.L Wolf grown in artificially polluted soils. J. Associ. Explor. Geochem..

[14] Fu G.L., Yang X.F., Han K.J., Xie H.C. (2021). Effects of exogenous selenium on physiological characteristics of *Salix babylonica* under 2,4-dinitrophenol stress. Pol. J. Environ. Stud..

[15] Shi X., Leng H.N., Hu Y.X., Liu Y.H., Duan H.P., Sun H.J., Chen Y.T. (2012). Removal of 2,4-dichlorophenol in hydroponic solution by four *Salix matsudana* clones. Ecotox. Environ. Safe..

[16] Xie H., Zhe W., Han K., Zhang T. (2020). Phytoremediation of Waste Water Containing Phenol by *Salix Matsudana* Seedlings and their Physiological Response. Glob. J. Bot. Sci..

[17] Riches M., Lee D., Farmer D.K. (2020). Simultaneous leaf-level measurement of trace gas emissions and photosynthesis with a portable photosynthesis system. Atmos. Meas. Tech..

[18] Yan F., Wang Q.L., Guo Y.Y., Zhang Y.J., Hou L.Y. (2016). Effect of NaCl stress on photosynthetic chlorophyll fluorescence characteristics of wild yellow rhizome leaves in Qilian Mountains. Northwest J. Botany.

[19] Ogunyemi S.O., Luo J., Abdallah Y., Yu S., Wang X., Alkhalifah D.H.M., Hozzein W.N., Wang F., Bi J., Yan C. (2024). Copper oxide nanoparticles: An effective suppression tool against bacterial leaf blight of rice and its impacts on plants. Pest Manag. Sci..

[20] Xue X., Wu Y.E. (2010). Determination of chlorophyll content of wheat leaves and its relationship with SPAD value. Hubei Agr. Sci..

[21] Thanuja V.S., Chandan R.S., Gurupadayya B.M., Prathyusha W., Indupriya M. (2014). Spectrophotometric determination of le vetiracetam using 2, 4-dnp in pharmaceutical dosage form. Indo Am. J. Pharm. Res..

[22] Li X.N., Yang Y.L., Jia L.Y., Chen H.J., Wei X. (2013). Zinc-induced oxidative damage, antioxidant enzyme response and proline metabolism in roots and leaves of wheat plants. Ecotox. Environ. Safe..

[23] Ying Y.Q., Song L.L., Jacobs D.F., Mei L., Liu P., Jin S.H., Wu J.H. (2015). Physiological response to drought stress in Camptotheca acuminata seedlings from two provenances. Front. Plant Sci..

[24] Yu S.J., Yang L.Y., Gao K.X., Zhou J.C., Lan X., Xie J., Zhong C.M. (2023). Dioscorea composita wrky5 positively regulates atsod1 and atabf2 to enhance drought and salt tolerances. Plant Cell Rep..

[25] Zhong J.Y., Zhang Y.L. (2013). Effect of low temperature on MDA content and electrical conductivity in branches of different apricot varieties. Tianjin Agr. Sci..

[26] Su P.Y., Zhang M.J., Wang S.J., Qiu X., Wang J.X., Du Q.Q., Guo R., Che C.W. (2020). Water sources of riparian plants based on stable hydrogen and oxygen isotopes in Lanzhou section of the Yellow River, China. J. Appl. Ecol..

[27] Liu Y., Miao H.T., Huang Z., Cui Z., He H.H., Zheng J.Y., Han F.P., Chang X.F., Wu G.L. (2018). Soil water depletion patterns of artificial forest species and ages on the *Loess Plateau* (China). Forest Ecol. Manag..

[28] Edward G.A., Forbes R., Johnston J.E., Archer A.R. (2017). SRC willow as a bioremediation medium for a dairy farm effluent with high pollution potential. Biomass Bioenerg..

[29] Zhou J.H., Cheng K., Zheng J.Y., Liu Z.Q., Shen W.B., Fan H.B., Jin Z.N. (2019). Physiological and biochemical characteristics of cinnamomum camphora in response to Cu-and Cd-contaminated soil. Water Air Soil Poll..

[30] Lavergne A., Voelker S., Csank A., Graven H., Boer H., Daux V., Robertson L., Dorado I., Sancho E., Battipaglia G. (2020). Historical changes in the stomatal limitation of photosynthesis: Empirical support for optimality. New Phytol..

[31] Pei B. (2013). Effect of Soil Water Stress on Photosynthetic Physiological and Biochemical Properties of Sea Buckthorn. Master’s Thesis.

[32] Alfonso P.M., Chiara M., Torres-Ruiz J.M., Jaume F., Fernández J.E., Luca S., Antonio D.E. (2014). Regulation of photosynthesis and stomatal and mesophyll conductance under water stress and recovery in olive trees: Correlation with gene expression of carbonic anhydrase and aquaporins. J.Exp. Bot..

[33] Vilyanen D., Pavlov I., Naydov I., Ivanov B., Kozuleva M. (2024). Peculiarities of dnpint and dbmib as inhibitors of the photosynthetic electron transport. Photosynth. Res..

[34] Jing T., Xie H.C., Sun J.W., Liu H.D., Li H. (2017). Tolerance and phytoremediation capacity of sunflower exposed to aniline wastewater. Acta Ecol. Sin..

[35] Farquhar G.D., Sharkey T.D. (1982). Stomatal conductance and photosynthesis. Annu. Rev. Plant Physiol..

[36] Shin Y.K., Bhandari S.R., Jo J.S., Song J.W., Lee J.G. (2021). Effect of drought stress on chlorophyll fluorescence parameters, phytochemical contents, and antioxidant activities in lettuce seedlings. Horticulturae.

[37] You X., Gong J.R. (2012). The significance of chlorophyll fluorescence kinetic parameters and example analysis. Western Forestry Sci..

[38] Zhang G.S., Hao L., Yan Z.J., Zhao X., Wang Y., Bai Y.R., Li X.L. (2017). The responses of chlorophyll fluorescence kinetics parameters of six tree species to soil moisture changes. J. Ecol..

[39] Khan S., Afzal M., Iqbal S., Khan Q.M. (2013). Plant–bacteria PAR tnerships for the remedi-ation of hydrocarbon contaminatedsoils. Chemosphere.

[40] Yan Q.Q., Zang J.S., Dai J.M., Dou Q.Q. (2019). Effect of betaine on photosynthesis and biomass accumulation of sea island cotton seedlings under salinity stress. J. Crop Sci..

[41] Chen H.X., Chen W., Jiang B.D., Gao H.Y., Zou Q. (2008). Effect of light-temperature cross-treatment on the activity and heat dissipation capacity of wheat violet yellows deoxygenase. J. Plant Ecol..

[42] Fu D.G., Zhang L.D., Li H., Li F., Yue Z.J., Li Y.B., Cai Q.C. (2023). Effects of the nitrogen form ratios on photosynthetic productivity of poplar under condition of phenolic acids. Int. J. Phytoremediat..

[43] Yuan L., Ali K., Zhang L.Q. (2005). Effects of NaCl stress on reactive oxygen metabolism and cell membrane stability in pistachio seedlings. Acta Phytoecol. Sin..

[44] Li Y.M., Guo X.W., Dai H.P. (2014). Physiological response of seedlings of Niu Zhanzu to salinity stress and its salt tolerance threshold. Northwest J. Botany..

[45] Mori I.C. (2004). Reactive oxygen species activation of plant Ca^2+^ channels. A signaling mechanism in polar growth, hormone transduction, stress signaling, and hypothetically mechanotransduction. Plant Physiol..

[46] Neto W.A.F., Dosselli R., Kennington W.J., Tomkins J.L. (2023). Correlated responses to selection for different cell size inchlamydomonas reinhardtiiusing divergent evolutionary pathways. J. Appl. Phycol..

[47] Zhang Z., Yu Y., Xi H., Zhou Y. (2021). Single and joint inhibitory effect of nitrophenols on activated sludge. J. Environ. Manag..

